# Comparison of postural sway in individuals with and without dynamic knee valgus

**DOI:** 10.1186/s13102-023-00686-4

**Published:** 2023-07-03

**Authors:** Kimia Karimi, Foad Seidi, Seyed Hamed Mousavi, Mohammad Alghosi, Nafiseh Homaie Morad

**Affiliations:** Department of Health and Sports Medicine, Faculty of Sports Sciences and Health, University of Tehran, Tehran, Iran

**Keywords:** Dynamic knee Valgus, Medial knee displacement, Postural control, Movement Variability, Balance

## Abstract

**Background:**

Dynamic knee valgus (DKV) is a multi-planar faulty movement pattern that can cause faulty postural control. The primary objective of this study is to investigate the differences in postural sway (PS) between individuals aged 18–30 years old diagnosed with and without DKV.

**Methods:**

In this cross-sectional study, 62 students (39 males and 23 females) with and without DKV (age: 24.58 ± 2.63 years) were selected and assigned to two groups by conducting the single-leg squat test in the screening stage. The Biodex balance system was then employed to compare the two groups in PS. Mann–Whitney U test was conducted to compare the groups in PS (p ≤ 0.05).

**Results:**

The study’s findings indicate that individuals with DKV did not exhibit any significant differences, compared to those without, about the anterior-posterior stability index (with p values for both static and dynamic situations at 0.309 and 0.198, respectively), medial-lateral stability index (with p values for both static and dynamic situations at 0.883 and 0.500, respectively), and overall stability index (with p values for both static and dynamic situations at 0.277 and 0.086, respectively).

**Conclusion:**

Though several possible factors could contribute to the lack of significant differences in postural sway between individuals with and without DKV, such as measurement tool differences, variable sensitivity in postural stability tests, and differences in movement variability and test stance, we recommend analyzing postural sway in more functional tasks and with different methodological patterns in future studies. Such research could help develop targeted interventions for individuals with DKV and offer a better understanding of the relationship between postural control and DKV.

## Background

Dynamic knee valgus (DKV) is a multi-planar lower limb faulty movement pattern. It is a combination of hip joint adduction, femoral anteversion, knee abduction, and external tibial torsion [1, 2]. DKV is more prevalent in women than men, and its risk increases from childhood to adolescence [3]. The dynamic version is considered a risk factor in lower limb injuries such as patellofemoral pain syndrome, anterior cruciate ligament injury, knee osteoarthritis, and iliotibial band syndrome [4]. The knee injuries of a lifetime are the significant causes of knee arthritis, particularly rheumatism, in advanced ages [5]. Moreover, knee valgus can be a secondary impairment due to several factors such as weakness of the hip abductor, increased femur anteversion, internal rotation of the tibia, a larger Q angle, and weak neuromuscular control over the hip [6, 7]. Control impairment in the sensorimotor system can change the neuromuscular system [8–10]. Such a neuromuscular change can lead to faulty movement patterns such as DKV during functional tasks. Functional neuromuscular control means the proper timing of muscles to produce adequate force and is necessary to create dynamic stability [11]. The adequate control of neuromuscular function in the trunk and pelvic complex plays a crucial role in preventing DKV [12–14]. Postural sway (PS) refers to the neuromuscular response that enables the maintenance of postural balance by causing motion in the body’s center of gravity within the base of support [15]. Therefore, it is imperative to ensure reasonable neuromuscular control in the trunk and pelvic complex to prevent DKV, and PS is a vital mechanism that facilitates postural stability. An impaired postural control can increase PS and also cause balance impairments [16]. Thus, postural stability and muscle activity could be measured to diagnose impaired proprioception and neuromuscular control [17]. There might also be a potential relationship between joint biomechanics and movement pattern changes and the knee valgus angle during functional activities [18]. An increase in the knee valgus angle affects the control of the center of pressure (COP). Hence, evaluating motor control has received a great deal of attention in preventing injuries, especially non-contact ones [19, 20].

Previous studies have analyzed kinematic variables and motor control in individuals with knee deformities, such as genu recurvatum, medial knee displacement, and genu varum [17, 19, 20]. Yazdani et al. [17] reported a significant difference between individuals with and without anterior-posterior (AP) or medial-lateral (ML) genu recurvatum in terms of motor control. Confirming the correlation between motor control impairments and knee deformities could stimulate additional research on postural control in individuals with knee deformities. Samaei et al. [20] found no significant differences in PS between women suffering from AP and overall genu varum and genu valgum and another unaffected group. However, there were significant differences in PS between those with and without ML deformities. That finding led to studies on motor control and PS in individuals with and without DKV. Vaz et al. [19] compared two groups of individuals with and without DKV in terms of motor control. Their results indicated that women with DKV had more random sways in the ML direction in the center of gravity and weaker balance. However, Mansouri et al. [21] reported that there were more variations in the COP of individuals without DKV than in those with DKV, and they also had weaker balance.

Furthermore, no studies have investigated PS differences in individuals with and without DKV. By analyzing postural sway, the study aims to determine if there is a difference in injury risk between the two groups, with potential implications for injury prevention. Additionally, the study expands on previous research on motor control in individuals with knee deformities by including non-athletes, who may be at higher risk due to weaker balance. The study’s findings could improve our understanding of evaluating and managing faulty movement patterns of the lower extremities. Given the significance of identifying risk factors to mitigate injuries, the purpose of this study was to evaluate the postural sway of individuals with and without DKV. We hypothesized that there is a significant difference in PS between individuals with and without DKV.

## Methods

### Research design

This study was a cross-sectional comparative analysis. Relevant ethical concerns described in the *Instructions of the Biomedical Research Ethics Committee of the University of Tehran* were taken into account, and an ethics code (IR.UT.SPORT.REC.1400.16) was obtained. Due to the COVID-19 pandemic during the research period (from September 1, 2021, to October 1, 2021), all participants were sent to the university’s health center before they came to the laboratory of the sports medicine department at the University of Tehran. The individuals were scheduled to be in the lab at exact appointments to avoid crowds and keep social distance all the time. The participants abided by the rules of no handshake, no touching of the eyes, mouth, and nose, and the use of gloves and disinfectants in all steps.

### Participants

The statistical population included male and female dormitory students of the University of Tehran aged 18–30. Using G-Power 3.1,  a priori power analysis to obtain 80% statistical power with α of 0.05, and effect size of 0.73 for a standard two-tailed hypothesis determined a total sample size of 62 participants [22]. The effect size was reported in previous studies ranging from 0.6 to 0.88 [23, 24]. Therefore, expecting an approximately 10% dropout rate, the target recruitment sample size was increased to 65. The participants were divided into two groups (33 individuals with DKV and 29 people without DKV) through a screening test. Participants were recruited for the study based on the following inclusion criteria: no history of regular exercise [25], with a body mass index (BMI) between 18 and 28 [25], absence of lower limb injuries or previous surgeries altering natural alignment (such as lumbar or lower limb surgeries), and no visual, vestibular, or neuromuscular impairments [17, 26], as well as no pain affecting movement strategies such as low back pain [27] or medical conditions associated with limited movement such as osteoarthritis [28]. The exclusion criteria included showing abnormal symptoms such as a body temperature of above 37° C and hypoxia before attending the laboratory (i.e., an oxygen level lower than 93%) and refusing to complete the tests in the scheduled session.

### Research instruments and procedures

**Fig. 1:**
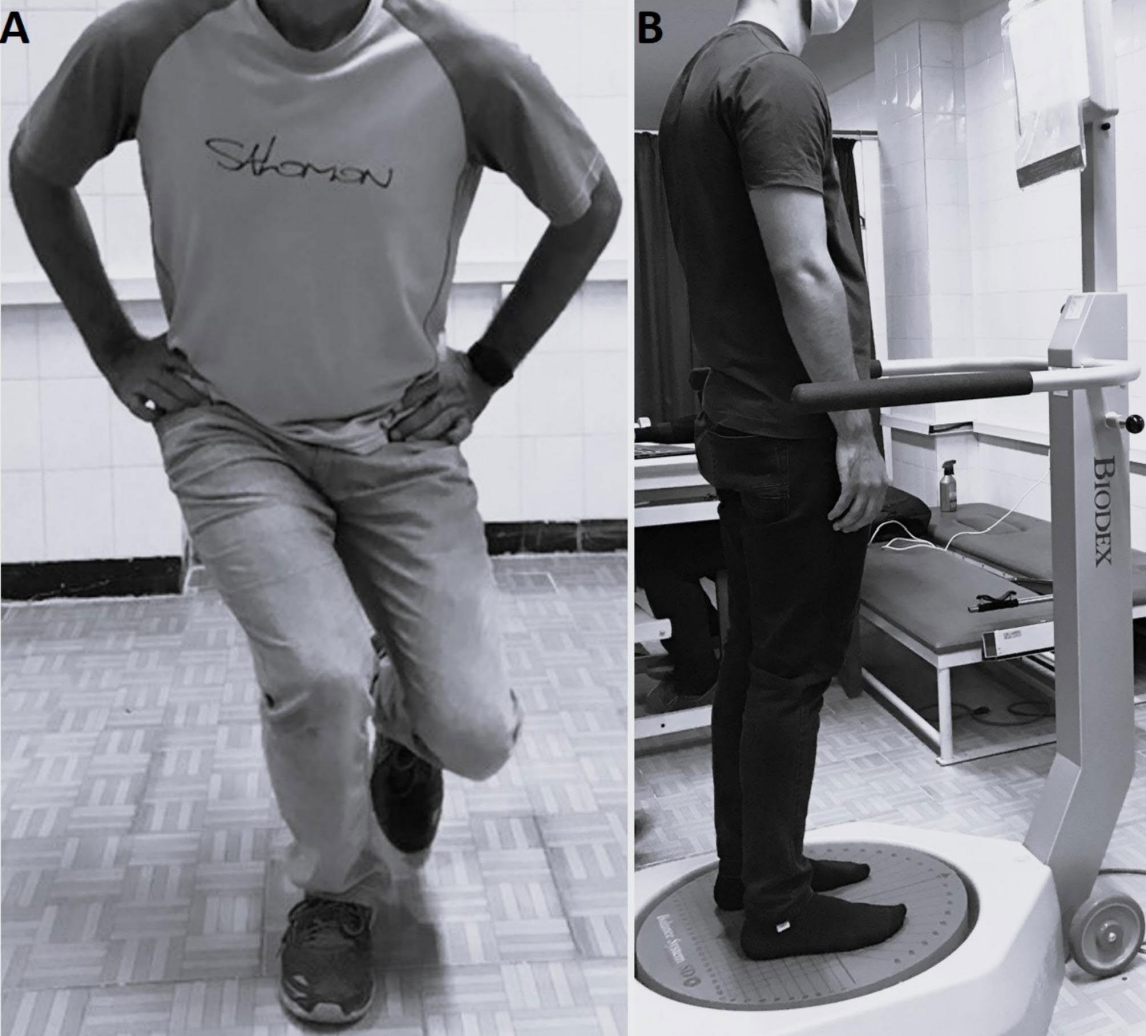
Single-leg squat test for screening the participants (**A**) and Biodex Balance System for evaluating postural stability (**B**)

The Biodex Balance System (BBS; Biodex Medical Systems, Shirley, NY, USA) was employed to evaluate static and dynamic balances (Fig. 1). This device can quantify the ability to maintain single-leg and double-leg postural stability, i.e., the stability of the foot on its area of contact, to distinct levels (from 1 to 12). The stability indices by the system describe the plane’s deviation from the horizontal position, including AP, ML, and overall stability indices. The corresponding reported reliability for those indices were 0.94, 0.95, and 0.93, respectively [29] and its validity as a balance evaluation instrument was then confirmed [30].

The single-leg squat test (SLST) was initially conducted to diagnose and screen participants for DKV (Fig. 1). The knee flexion angle was nearly 60º during the test [25]. Moreover, the single-leg squat clinical test examines the alignment of the lower limb. It is employed to diagnose faulty trunk, hip, and lower limb movement patterns. The test had acceptable validity and excellent interrater reliability (ICC: 0.97–0.99 ) [31, 32].

Sixty-five eligible participants completed the personal information forms consisting of different items on vestibular and visual medical issues, neuromuscular disorders, medical history, employment conditions, and previous injuries. The initially selected individuals were asked to attend the Department of Sports Medicine laboratory at the University of Tehran. The session began by measuring the weight and height of participants to calculate BMI. After that, a specialist in corrective exercises examined participants for postural deformities. The eligible individuals were informed of the exact research process according to inclusion and exclusion criteria. They also filled out consent forms. They were then asked to put on appropriate comfortable clothes and their own sports shoes [33], warm up for five minutes consisting of jogging and stretching exercises [34], and be prepared for a functional SLST for initial diagnosis and screening for DKV by the examiner. Following a warm-up and information session on the test procedure, each individual performed five single-leg squats on their dominant leg with a knee flexion angle of nearly 60º. DKV was diagnosed when the midpoint of the patella was moved inward to a point past the big toe in three out of five repetitions. Each repetition’s two-dimensional correctness was controlled both visually and by recording the trials with a digital camera [25]. Moreover, a 60-beat-per-minute metronome was utilized to control the speed. Each participant would go down during the first two clicks and return to the original position in the next two. The entire process was recorded on a digital camera. The participants would receive no feedback on the implementation of trials and synchronize the test by using the metronome clicks. Ultimately, individuals with and without DKV were divided into two separate groups. The test-retest reliability for the pilot study was 0.92, and PS was measured in all directions for the participants diagnosed with DKV through the Biodex device. They did the static and dynamic postural stability tests to measure the PSs (i.e., overall, AP, and ML). The test procedure and operation of the device were explained to the participants, and an examiner adjusted the height of the handles and display, the required stability level, the duration of the test, and the rest time for each participant. The participants then stood on the instrument with their feet apart as wide as their shoulders and their eyes open. The stability level of the device was set at a static state for the static test and 8 for the dynamic test. The actual test was performed after participants were familiar with the test. Each test was repeated three times in each state in 20 s with a 10-second rest time between repetitions. The average sway index in three repetitions was recorded as the participant’s score. A higher stability index indicated weaker postural stability. All participants underwent PS evaluations during the same time-period session, which was from 10:00 am to 12:00 pm. The test-retest reliability for this test was 0.92 in a pilot study.

### Statistical analysis

The individuals with and without DKV were compared in terms of the measured parameters in IBM SPSS (IBM Corp. Released 2021. IBM SPSS Statistics for Windows, Version 28.0. Armonk, NY: IBM Corp). The means and standard deviations of all variables were also calculated. Before conducting inferential statistical analysis, a normality check was performed using the Shapiro-Wilk test. As the results indicated a deviation from a normal distribution, the Mann-Whitney U test was employed to accurately compare the different groups in the study. The alpha value for the 95% significance level was set at 0.05.

## Results

A total of 62 students participated in the study. The demographic data of all participants are summarized in Table 1. There were no significant differences between the individuals with and without the DKV group regarding the participants’ age, height, weight, and BMI (p > 0.05).

**Table 1:** The characteristics of the participants

Variable	Individuals with DKV	Individuals without DKV	P-value	Total
**Age (year)**	24.48 ± 2.86	24.69 ± 2.39	0.381	24.58 ± 2.63
**Height (cm)**	173.85 ± 10.70	174.90 ± 8.79	0.339	174.34 ± 9.83
**Weight (kg)**	68.17 ± 9.61	69.67 ± 7.62	0.251	68.87 ± 8.70
**BMI (kg/m** ^**2**^ **)**	22.51 ± 2.25	22.80 ± 2.33	0.311	22.65 ± 2.27
**Gender n (%)**	Men: 19 (57.6) Women: 14 (42.4)	Men: 20 (69.0) Women: 9 (31.0)	NA	Men: 39 (62.9) Women: 23 (37.1)

**Notes**: Data was provided as means with standard deviation. The P-value is calculated using an independent t-test for comparing ∆ between individuals with and without DKV.

**Abbreviations**: DKV: dynamic knee valgus, cm: centimeters, kg: kilograms, BMI: body mass index, m: meter, NA: not applicable.

Table 2 reports the descriptive information of research variables and the results of the between-group analysis of variables in the Mann–Whitney U test. No statistical differences between the groups were found in PS outcomes (P-value for APSI in static and dynamic situations, respectively: 0.309, 0.198), (P-value for MLSI in static and dynamic situations, respectively: 0.883, 0.500), and (P-value for OSI in static and dynamic situations, respectively: 0.277, 0.086).

**Table 2:** Descriptive information of variables and results of the Mann–Whitney U test

Variable	Individuals with DKV	Individuals without DKV	P-value	ES (d)	MD (95% CI)	Observed power
Static platform						
APSI (degree) MLSI (degree) OSI (degree)	1.78 ± 1.52 1.10 ± 1.10 2.32 ± 1.63	1.28 ± 0.78 0.96 ± 0.67 1.76 ± 0.86	0.309 0.883 0.277	0.41 0.14 0.42	0.49 (-0.1-1.1) 0.12 (-0.3-0.6) 0.56 (-0.9-1.2)	0.350 0.084 0.374
Dynamic platform						
APSI (degree) MLSI (degree) OSI (degree)	1.32 ± 0.96 0.88 ± 0.97 1.72 ± 1.37	1.43 ± 0.67 0.86 ± 0.49 1.81 ± 0.70	0.198 0.500 0.086	-0.12 0.03 -0.08	-0.10 (-0.5-0.3) 0.02 (-0.3-0.4) -0.08 (-0.6-0.4)	0.076 0.051 0.060

**Notes**: Data was provided as means with standard deviation. The P-value is calculated using the Mann–Whitney U test for comparing PS between individuals with and without DKV. Effect size (Cohen`s d: the standardized mean difference between two groups): 0.2 = small; 0.5 = medium; 0.8 = large

**Abbreviations**: DKV: dynamic knee valgus, APSI: anterior-posterior sway index, MLSI: medial-lateral sway index, OSI: overall sway index, ES: effect size, d: cohen’s d, MD: mean differences, 95% CI: 95% confidence interval

## Discussion

This study aimed to compare individuals with and without DKV in terms of PS. The results indicated no significant differences between the two groups in AP, ML, and overall PS. The corresponding (static, dynamic DKV) p-values were (0.309, 0.198), (0.883, 0.500), and (0.277, 0.086), respectively. Several possible reasons can justify those results. The research literature suggests that the following factors could be the possible causes.

The first possible factor is the difference in PS measurement tools. Mansouri et al. [21] indicated that the DKV group experienced more variations in COP, a finding which is inconsistent with our results. The PS variables in that study were more strongly related due to their larger effect size (η_p_^2^ = 0.457). They used a Kistler force plate (KFP) instead of the BBS used in this study. The time to stability is among the most used criteria for evaluating dynamic stability in athletes. Measuring that value by KFP is a widely used, effective method for assessing the conditions of athletes and providing information for coaches and specialists. However, posturography devices such as BBS evaluate postural control in relatively static conditions. They are widely used by non-athletes and clinic clients [35]. KFP is considered the gold standard in collecting kinetic information [36]. Using this standard in other studies might be the source of discrepancy between their results. BBS consists of a moving balance platform that produces up to 20º of planar slope in a 360º arc of motion. It reports the AP, ML, and overall stability indices in degrees. Higher degrees indicate lower stability levels. In contrast, KFP tracks COP variables such as maximum and average sway radius, maximum posterior, maximum left, and maximum right sways, and the total length of the sway path. It reports the measurements in millimeters [37]. The participants in the other study were female athletes, whereas “not having a regular exercise history” was an inclusion criterion in our study. Hence, another reason for differences between their results and ours could be the recruitment of various participants and the use of diversely assigned tasks, as the neuromuscular functionality differs in athletes and non-athletes. The biomechanical analysis of the landing task in their study might provide more precise evidence in the preventive exercise program, as the single-leg landing task is more similar to the athletes’ functions in sports than the task of maintaining double-leg postural stability. The research literature [35] recommends using BBC for measuring balance in mostly non-athlete populations, something which results in no significant differences between individuals with and without DKV in terms of PS. As further studies are published in this area, a different result might be produced by performing the task in a dynamic state (similar to daily activities). Comparing individuals with and without DKV in terms of PS through a double-leg stability test on BBS cannot produce significantly different results in dynamic and static situations.

The second possible explanation for the lack of significant difference between the two groups in PS could be the sensitivity of the postural stability test variable [17]. Static postural stability tests might include limited joint information [38]. This test involves a fixed platform, whereas a dynamic test involves a moving one, which might emulate the actual life activities more accurately. However, it still needs to be a functional test as evaluating dynamic balance and stability must include functionally more relevant tasks. Such tasks represent regular dynamic actions in a particular activity [39]. The results were not unexpected as the test was not conducted in a functional state more similar to daily activities. Hence, the postural stability might not be sensitive enough to determine differences between individuals with and without DKV.

Movement variability (MV), which is defined as natural differences in motor control strategies applied in different actions, could be another cause of discrepancies between the results [40]. Although movement consistency (MC) is essential in preventing risky biomechanics (e.g., DKV), MV has a vital role in preventing injuries. MV is a crucial indicator of appropriate adjustment in complex systems [41]. Thus, it should be included in movement-based screening followed by evaluating the movement stability every few repetitions. For example, individuals scoring three in a functional movement screening test will not do the same test in the later repetitions. The score could mean that the individual is skillful at that movement, whereas later test repetitions do not show MC [42]. Our study only evaluated three PS test repetitions. Assessing PS in more repetition could have produced different results due to MV and a significant difference between the test and control groups. Brown et al. [43] compared a group with a history of an ankle injury with a control group in terms of MV. Those with a history of injury had higher levels of MV than the control group. The neuromuscular difference in individuals suffering from ankle instability might partially be due to central nervous system-induced motor control changes, leading to MV. Individuals with DKV might have been using more diverse strategies than those without it due to higher levels of MV and, thus, less MC. In other words, the average PS of the group with DKV would not be consistently compared with the same variable in the other group, and no significant differences shall be formed between the groups.

Another factor that might be contributing to the absence of a statistically significant difference between the individuals with and without DKV could be attributed, in part, to gender differences in kinematic chains, especially those related to knee alignment [44–46]. Previous studies have indicated that women tend to exhibit more knee valgus and extension angles during maneuvers that involve cutting, jumping, and landing, compared to men [44, 45]. These biomechanical differences may place women at an elevated risk of suffering knee injuries, such as tears to the anterior cruciate ligament [12]. Although our study did not include separate analyses of male and female participants, we acknowledge that gender disparities in lower limb kinematics could have influenced the research outcomes.

The last possible factor is the single-leg versus double-leg stance PS test for diagnosing individuals with DKV. According to Vaz et al. [19], the PS and COP variations in ML directions were not significantly different between a group with medial displacement of the knee and a control group. However, their results indicated a significant difference in the AP type of deformity, a finding which was inconsistent with our results. This can be justified as the single-leg standing reduced the AP support surface, requiring more control of the center of mass. This study measured the PS in a double-leg standing stance where it was easier to maintain stability. Maintaining single-leg stability was a more limited task as the support surface was limited to the area under the foot or shoe. This mechanical limitation reduced the somatosensory information of the sole used for maintaining balance. Maintaining balance in the single-leg stance also was more difficult due to intricate reactive control strategies endured by proximal and distal muscles [47]. The above differences between single-leg and double-leg tests explained the differences between our and their results.

### Limitations

Considering the tests conducted in our study, the nature of DKV, and the lack of a significant difference in PS variables between groups with and without DKV, using a more functional test to compare postural control between the groups would have provided better results. Clinical studies are prone to examiner bias even with high test-retest reliability (0.92) in the SLST. The gold standard in the kinematic evaluation of DKV is three-dimensional single-leg movement analysis [39]. Our study did not perform such an analysis due to the unavailability of the necessary instrument. We suggest that kinematic research in individuals with DKV be conducted by executing more functional single-leg tasks such as walking on the gold-standard tool (e.g., KFP or Inertial Measurement Units).

## Conclusions

A comprehensive understanding of the relationship between DKV and postural stability requires careful consideration of several important factors. These factors include differences in measurement tools, variable sensitivity in postural stability tests, and discrepancies in movement variability and test stance. To gain greater insight into the impact of DKV on postural stability, it is also necessary to consider a wider range of functional tasks. In essence, a more sophisticated approach is required to effectively investigate the correlation between DKV and postural stability to facilitate a comprehensive understanding of the topic. Hence, our study attempts to address these key considerations to gain a clearer and more robust understanding of the relationship between DKV and postural stability, which can ultimately inform clinical assessments and treatment approaches.

## Data Availability

The datasets generated and/or analyzed during the current study are available on request from the corresponding author.

## Abbreviations

DKV: Dynamic Knee Valgus
PS: Postural Sway
COP: Center of Pressure
BMI: Body Mass Index
CM: Centimeters
KG: Kilograms
M: Meter
APSI: Anterior-Posterior Sway Index
MLSI: Medial-Lateral Sway Index
OSI: Overall Sway Index
BBS: Biodex Balance System
SLST: Single-leg Squat Test
MV: Movement Variability
MC: Movement Consistency
KFP: Kistler Force Plate
